# Assessment of exposure determinants and exposure levels by using stationary concentration measurements and a probabilistic near-field/far-field exposure model

**DOI:** 10.12688/openreseurope.13752.1

**Published:** 2021-06-21

**Authors:** Antti Joonas Koivisto, Andrea Spinazzè, Frederik Verdonck, Francesca Borghi, Jakob Löndahl, Ismo Kalevi Koponen, Steven Verpaele, Michael Jayjock, Tareq Hussein, Jesus Lopez de Ipiña, Susan Arnold, Irini Furxhi

**Affiliations:** 1 Air Pollution Management, Willemoesgade 16, st tv, Copenhagen, DK-2100, Denmark; 2 ARCHE Consulting, Liefkensstraat 35D, Wondelgem, B-9032, Belgium; 3 Institute for Atmospheric and Earth System Research (INAR), University of Helsinki, PL 64, Helsinki, FI-00014 UHEL, Finland; 4 Dipartimento di Scienza e Alta Tecnologia, Università degli Studi dell’Insubria, via Valleggio 11, Como, IT-22100, Italy; 5 Division of Ergonomics and Aerosol Technology, Lund University, Lund, SE-22100, Sweden; 6 FORCE Technology, Copenhagen, DK-2605, Denmark; 7 Nickel Institute, Rue Belliard 12, Brussels, B-1040, Belgium; 8 Belgian Center for Occupational Hygiene, Technologiepark 122, Zwijnaarde, B-9040, Belgium; 9 Jayjock Associates, LLC, 168 Millpond Place, Langhorne, PA, USA; 10 Department of Physics, The University of Jordan, Amman, 11942, Jordan; 11 TECNALIA Research and Innovation - Basque Research and Technology Alliance (BRTA), Parque Tecnológico de Alava, Leonardo Da Vinci 11, Miñano, 01510, Spain; 12 School of Public Health, University of Minnesota, 420 Delaware St SE, Minneapolis, MN, USA; 13 Department of Accounting and Finance, Kemmy Business School, University of Limerick, Limerick, V94 T9PX, Ireland; 14 Transgero Limited, Cullinagh, Newcastle West, Co. Limerick, Limerick, V42 V384, Ireland

**Keywords:** Conditions of Use, inhalation exposure, probabilistic model, stationary measurements, EN 689, ECHA, REACH

## Abstract

**Background:** The Registration, Evaluation, Authorization and Restriction of Chemicals (REACH) regulation requires the establishment of Conditions of Use (CoU) for all exposure scenarios to ensure good communication of safe working practices. Setting CoU requires the risk assessment of all relevant Contributing Scenarios (CSs) in the exposure scenario. A new CS has to be created whenever an Operational Condition (OC) is changed, resulting in an excessive number of exposure assessments. An efficient solution is to quantify OC concentrations and to identify reasonable worst-case scenarios with probabilistic exposure modeling.

**Methods:** Here, we appoint CoU for powder pouring during the industrial manufacturing of a paint batch by quantifying OC exposure levels and exposure determinants. The quantification was performed by using stationary measurements and a probabilistic Near-Field/Far-Field (NF/FF) exposure model. Work shift and OC concentration levels were quantified for pouring TiO _2_ from big bags and small bags, pouring Micro Mica from small bags, and cleaning. The impact of exposure determinants on NF concentration level was quantified by (1) assessing exposure determinants correlation with the NF exposure level and (2) by performing simulations with different OCs.

**Results: **Emission rate, air mixing between NF and FF and local ventilation were the most relevant exposure determinants affecting NF concentrations. Potentially risky OCs were identified by performing Reasonable Worst Case (RWC) simulations and by comparing the exposure 95 ^th^ percentile distribution with 10% of the occupational exposure limit value (OELV). The CS was shown safe except in RWC scenario (ventilation rate from 0.4 to 1.6 1/h, 100 m ^3^ room, no local ventilation, and NF ventilation of 1.6 m ^3^/min).

**Conclusions:** The CoU assessment was considered to comply with European Chemicals Agency (ECHA) legislation and EN 689 exposure assessment strategy for testing compliance with OEL values. One RWC scenario would require measurements since the exposure level was 12.5% of the OELV.

## Plain language summary

Worker’s inhalation exposure is defined by process emissions, dilution and mixing of concentrations, worker exposure time and personal protective equipment. Exposure determinants define the exposure level, which are related to i) the process emissions such as process parameters and materials and integrated emission controls, ii) environmental conditions such as dilution and removal of pollutants by ventilation and iii) workers behavior such as exposure duration. Here is presented how a probabilistic exposure model taking into account exposure determinant randomness can be used to quantify the exposure factors effect on the workers exposure. This can be used to set evidence-based conditions of use for safe work and justify the need for external emission and exposure controls or personal respiratory protective equipment. The method was applied to predict paint factory worker exposure to inorganic dust during pouring of TiO _2_ and Micro Mica powders and setting conditions of use for safe working. The method was found to comply with the Registration, Evaluation, Authorization and Restriction of Chemicals (REACH) chemical safety assessment and EN 689 basic exposure characterization.

## Introduction

**Figure 1.**
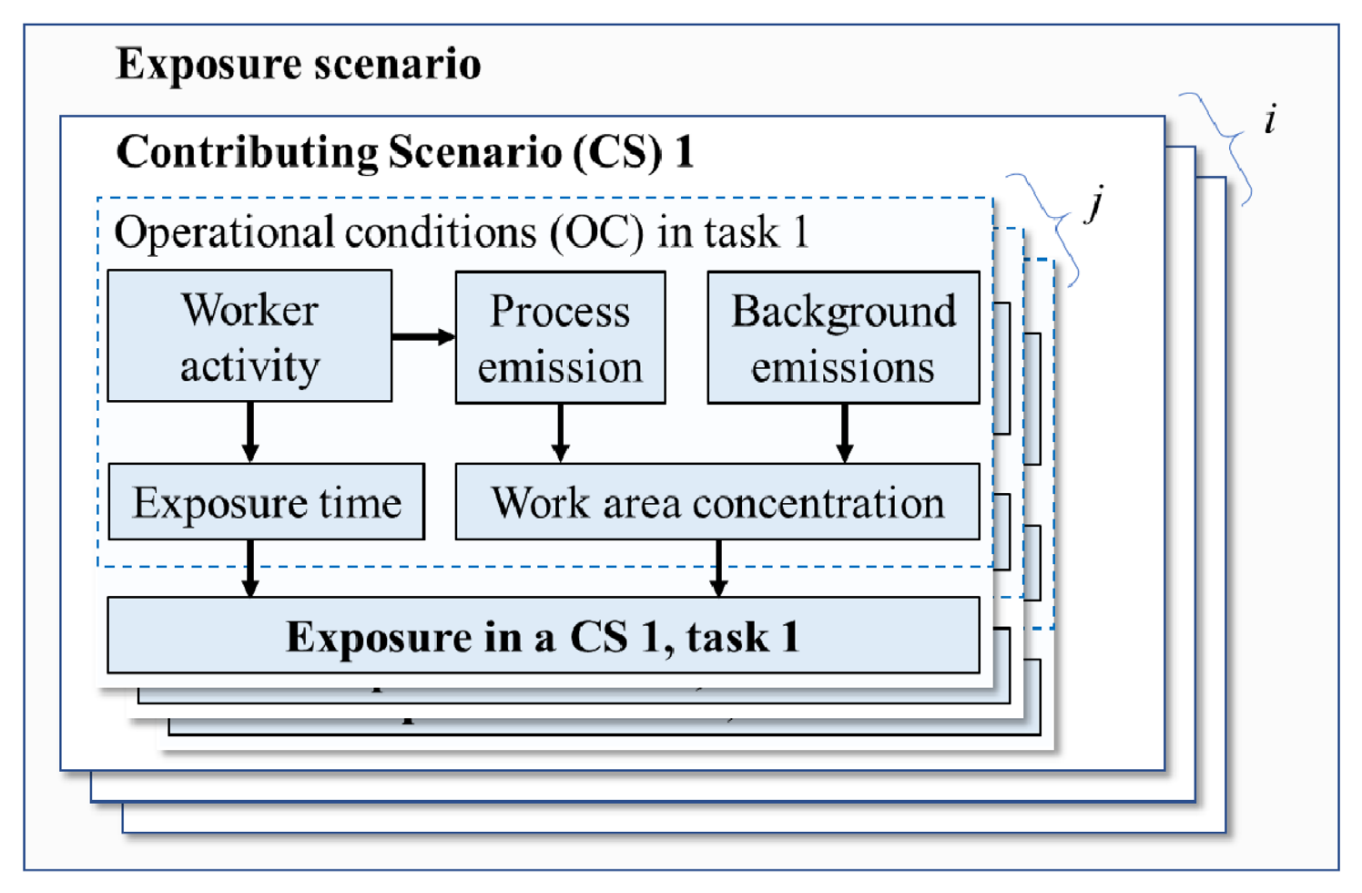
A simplified concept of an occupational exposure scenario consisting of i Contributing Scenarios (CSs) with j Operational Conditions (OCs). The relations between different factors affecting on the personal exposure level are shown with arrows.

In the life-cycle of a substance, the Operational Conditions (OCs) and risk management measures must be determined for each of the identified uses ( ECHA, 2016). Chemical manufacturing and product formulation consist of many steps ( Litster & Bogle, 2019), where each step can contain one or more Exposure Scenarios (ESs). An ES consists of *i* ( *i* ≥ 1) Contributing Scenarios (CSs), where each CS can have *j* ( *j* ≥ 1) work tasks with different OCs ( Figure 1). In this study, the European Chemicals Agency (ECHA) terminology was used, see Text S1, *Extended data* ( Koivisto *et al*., 2021).

The exposure in a CS can be measured by personal or stationary samplers or it can be estimated/predicted using exposure models ( ECHA, 2016). Breathing zone measurement is the aggregation of all factors related to personal exposure, making it the most precise technique for quantifying personal worker exposure. A drawback is that the causes of exposure are challenging to assess due to personal factors. Stationary measurements are related to the personal exposure, but the relation depends on the sample location representativeness, among other factors ( Cherrie, 2004; Esmen & Hall, 2000). To translate the work area concentration to personal exposure over a full work shift requires concentration measurements from each performing task and the exposure durations. In addition, the relation between stationary and worker breathing zone measurements has to be quantified if stationary measurements are applied for personal exposure assessment ( Lange, 2003).

Registration, Evaluation, Authorization and Restriction of Chemicals (REACH) legislation requires quantitative exposure estimates for all CSs unless qualitative assessment can be argued as stated in ECHA chapter R.14 ( ECHA, 2016). A new CS has to be created whenever a condition ( *e.g.*, tasks, task frequencies or durations, process parameters or environmental conditions) is changed. ECHA R.14 specifies that “ *When the assessment is based on measured data, it is often the case that these measured data have been collected across several different tasks over a shift. In this case, the contributing activities that are relevant for the exposure scenario must still be described one by one, even if it is not possible to identify data points from the measured data set that are applicable to individual contributing activities. If the conditions are the same across all tasks, the contributing activities may be linked to one set of use conditions, which correspond to the conditions that are represented by the measured exposure data (covering both routes of exposure).”* According to authors interpretation, this means that exposure needs to be assessed for each CS and each OC separately.

Conditions of Use (CoU) describe the OCs and Risk Management Measures (RMMs) that are appropriate to maintain exposure at a safe level. CoU need to be reported when a substance’s hazard is identified, and it is used to communicate conditions/measures for safe use in the supply chain. CoU can be defined as those leading to risks in each CSs and OCs that are characterized as acceptable. Setting the CoU for an exposure scenario requires risk assessment of all relevant combinations of CSs and OCs. Thus, the total number of exposure assessments needed to set CoU should include all relevant combinations of *OC _i,j_* for a single ES ( Figure 1), leading to a high number of assessments.

Exposure determinants that need to be reported with CoU can be separated into three categories:

1) Process emissions (flow rate, energy level, emission controls, *etc.*),
2) Environmental conditions (dilution, removal, background concentration, *etc.*) and
3) Personal behaviors (exposure time in different areas, carefulness, experience, *etc.*).

All exposure determinants are physical observables that can be quantified to form and improve a model structure and parametrization (see Text S2, *Extended data* ( Koivisto *et al*., 2021); Jensen *et al*., 2018). ECHA R.14 gives examples of the CoU information requirements. Exposure determinants relevance (sensitivity) varies significantly depending on OCs (Text S2, *Extended data* ( Koivisto *et al*., 2021)) and it usually needs to be assessed for each OC. Frequently, the exposure level depends on individual’s working practices, such as carefulness, that reduces exposure level ( Jensen *et al*., 2015) or efficiency that reduces the exposure at the same processing volumes. These factors need to be considered when setting the appropriate CoU for an ES.

The workers often combine more than one task and rotate between workstations. The planning and organization of how workers are assigned to a specific task during a day also varies per company. Varying tasks, exposure durations among other variable exposure determinants makes the exposure assessment probabilistic in nature which means that exposure conditions cannot be determined with individual exposure measurement. CoUs for a specific CS or OC are challenging to establish by using personal monitoring data since a high number of personal exposure measurements are needed in order to cover all relevant work shift combinations in one ES.

An alternative method is to quantify Reasonable Worst Case (RWC) concentrations for each OC and then predict the probabilistic exposure distribution based on workers’ behavior. The exposure risk in a work shift is usually limited by the exposure time that is usually set as 8-h. With probabilistic simulations, all OC combinations that can lead to excess risk during a work shift, can be precisely identified. Individual OC contributions to work shift exposure level can be quantified under RWC conditions or as the highest theoretical value. This can be used to justify the OC risk level, which is a key factor in efficient risk communication. The number of personal exposure measurements can be reduced by focusing on the potentially risky OCs. The probabilistic exposure assessment can quantify relevant exposure determinants having a high impact on the exposure level, which are essential components in efficient risk management and communication.

In this study, we specify CoU for powder pouring during industrial manufacturing of a paint batch by using measured work area concentrations and probabilistic exposure modeling. We demonstrate *i*) how to parameterize a probabilistic Near-Field/Far-Field (NF/FF) exposure model, *ii*) how to simulate different OCs to quantify exposure determinants impact on the exposure level and *iii*) how to specify CoU.

## Methods

The modeling is performed by using a probabilistic NF/FF model Task Exposure Assessment Simulator (TEAS) version 1.0 (2019) ( Exposure Assessment Solutions, Inc., Missouri, U.S.). TEAS is similar to IH Mod 2.0 for Microsoft Excel, which can be used as a freely available alternative. The NF/FF model theoretical background and different constructions are described by Ganser & Hewett (2017). TEAS provides statistical evaluation of the exposure distribution and sensitivity charts for determining which task contributes most to the average exposure and which task contributes most to variability and a task sensitivity analysis is to identify the exposure determinant that have the greatest effect on exposure variability, and whether or not that effect is positive or negative. Spearman correlation is calculated to describe the task and/or exposure determinant correlation with the concentration level.

The model application is demonstrated by using published datasets considering workers’ exposure to respirable particles in a paint factory ( Fonseca *et al*., 2021; Koivisto *et al*., 2015). The model is used to predict worker’s inhalation exposure during a paint batch manufacturing consisting of three pouring tasks and a virtual cleaning phase (see day 1 in Koivisto *et al*., 2015). The aforementioned article reports NF, FF and worker breathing zone respirable mass concentrations during the pouring of TiO _2_ and Micro Mica powders into a tank.

Koivisto *et al*. (2015) used DustMonitor NF measurements to calculate emission rates in mg/min by using a deterministic NF/FF model (Text S3, *Extended data* ( Koivisto *et al*., 2021)). The air mixing flow rate between NF and FF, *β* (m ^3^/min), calculation methods are described by Ganser & Hewett (2017). Text S4, *Underlying data* ( Koivisto *et al*., 2021) gives a brief summary of the NF/FF model and the *β* approximation methods. Here, we tested and approximation method assuming low FF concentration and without emissions from the NF source where the *β* can be calculated from the NF concentration decay.

**Figure 2.**
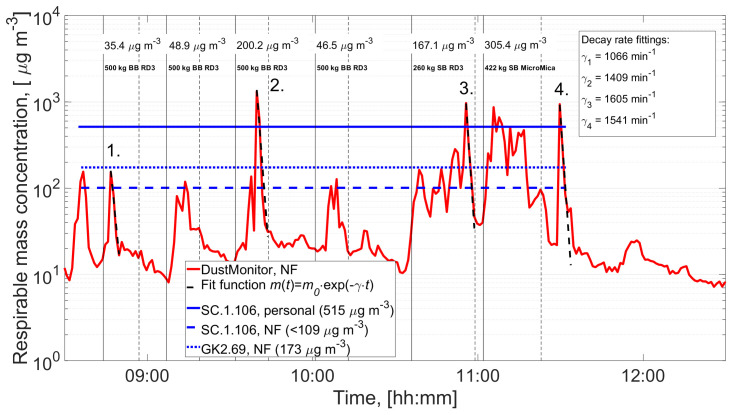
Near-Field (NF) respirable mass concentration measured by the NF DustMonitor at 1 min intervals (particle density 1.7 g/cm ^3^). Solid and dashed black lines show the pouring process start and end times, respectively. Average mass concentrations during pouring tasks are given over the pouring duration. Fitting for exponential concentration decay was performed for concentration peaks 1 to 4 to evaluate the NF ventilation rate (Text S2, Extended data ( Koivisto *et al.*, 2021)). Gravimetrical personal and NF samplers show the mean mass concentration level defined for the sampling time intervals specified with blue lines. The figure is modified from ( Koivisto *et al.,* 2015).

Figure 2 shows the DustMonitor concentrations with average task concentrations measured during the pouring. The particle average density was selected so that the DustMonitor respirable mass concentration corresponds to the average mass concentration measured by the NF gravimetric sampler GK2.69 over the respective time period ( Koivisto *et al*., 2015). In their gravimetric analysis sampling cassette wall losses were not evaluated, which can be significant for conductive cassettes as used by Koivisto *et al*. (2015). The Wall losses of SC.1.106 and GK2.69 sampler is not reported but for other respirable samplers’ using conductive cassettes the median wall losses is shown to vary from 5% to 56% with a maximum loss varying from 12% to 93% ( Harper, 2020). In Koivisto *et al*. (2015) study, 95% of the respirable mass was in the size range from 0.6 to 13.1 μm which reduces the wall losses by electrophoresis. In this study, the losses were not taken into account because it should be evaluated by using the occupational aerosol.

Because Koivisto *et al*. (2015) used 1 s time resolution and TEAS uses 1 min time resolution, the emission rates were adjusted so that the total mass release is the same. The contextual information described by Koivisto *et al*. (2015) and Fonseca *et al*. (2021) is used to set the default values for the exposure determinants.

The paint factory was naturally ventilated and there was no functioning Local Exhaust Ventilation (LEV) at the pouring station. Fonseca *et al*. (2021) shows a layout of the pouring hall (see Figure 1 in their study), the pouring station and an example of TiO _2_ powder pouring from a big bag (see Figure S1c, in the Supporting Information in their study). The powders were poured into the mixing tank from a 0.8 m × 0.8 m opening. The NF sampling location was ca. 0.5 m from the opening at the left-hand side of the worker at chest height. The modelled work shift duration was 160 min and it consisted of four tasks:

1. Pouring 2000 kg TiO _2_ (CAS: 13463-67-7) from big bags (4×500 kg);
2. Pouring 260 kg TiO _2_ (CAS: 13463-67-7) from small bags (10×25 kg + residual);
3. Pouring 422 kg Micro Mica (CAS: 12001-26-2) from small bags (17×25 kg + residual);
4. Simulated cleaning without emissions.

Simulations were performed for 10,000 scenario iterations. The room volume *V _room_* and air exchange rate *AER* were generated for the first box model task and were used for the following box model tasks as well ( *i.e.*, *V _room_* and *AER* remained constant over the remaining work shift).

Model parametrization was done using either the measured or reported values as mean values and the range estimated individually according to Table 1. Parameter ranges were set according to the best knowledge and observations by Koivisto *et al*. (2015) and Fonseca *et al*. (2021). Task simulation time was set to correspond to the measured process time within the model time resolution of 1 min. Here we focused on the concentration related to the pouring process where background concentrations were assumed to be insignificant.

**Table 1.** Parametrization of the work environment and tasks used for probabilistic exposure modeling. Parameter ranges were set according to the best knowledge, and observations by Koivisto *et al*. (2015) and Fonseca *et al*. (2021). The individual input parameters used in each modelled exposure scenario are available in Appendix S1 to S9 in Underlying data ( Koivisto *et al*. 2021).

Work environment (same for all tasks)		
Parameter	Value	Comments
Room volume, *V _room_* (m ^3^)	Linear range: 1300 to 1500 m ^3^	Empty hall volume is 1500 m ^3^, actual volume depends on storage loading.
General ventilation air exchange rate, *AER* (1/h)	Linear range: 2 to 8 1/h	Natural ventilation rate reported being 5 1/h based on expert estimate ( Koivisto *et al*., 2015). The range of the ventilation rate was set high due to large uncertainty.
Near-Field (NF) volume, *V _NF_* (m ^3^)	Linear range: 1 to 2 m ^3^	Partially covered pouring station. NF volume is assumed to range from 1 to 2 m ^3^ covering the pouring process, NF instruments and breathing zone. The same NF volume was used for the big bag and small bag pouring even though the worker is closer to the source in small bag pouring. Note, Koivisto *et al*. (2015) used a NF volume of 8 m ^3^ which is high for the partially enclosed NF volume.
Air mixing flow rate between NF and Far-Field (FF), *β* (m ^3^/min)	Triangular distribution with: Min: 0.649 m ^3^/min Mode: 3.2 m ^3^/min Max: 10 m ^3^/min	Specified for a cube with side length from 1 to 1.25 m (1 to 2 m ^3^) and both sides and back closed by a curtain. The random air speed was defined by using Baldwin & Maynard (1998) according to Text S4, *Underlying data* ( Koivisto *et al*., 2021). The flow rate was calculated from the random air speed and one half of the near-field volume free surface ( Keil & Zhao, 2017).
Task 1: Pouring 4×500 kg TiO _2_ from big bags		
Parameter	Value	Comments
Repetitions per shift, *n* (-)	4	Four big bags.
Pouring duration per bag, *t _pouring_* (min)	Linear range: 21 to 25 min	Measured as the range for start of the pouring to changing the bag to new.
Emission rate, *G* (mg/min)	Triangular distribution with: Min 0.43 mg/min Mode 0.87 mg/min Max 1.74 mg/min	The mean value was calculated from NF respirable mass concentration (Text S3, *Extended data*). The minimum value was set to 0.5 × mean *G* and maximum 2 × mean *G*. The multipliers are based on a personal judgement and typical NF/FF model precision ( Jayjock *et al*., 2011).
Pouring time (emission time), *t _G_* (min)	Linear range: 11 to 13 minutes	The big bag is emptied within 11 to 13 minutes.
Task 2: Pouring 10×25 kg TiO _2_ from small bags		
Parameter	Value	Comments
Repetitions per shift, *n* (-)	10	Ten small bags. Residual is not included.
Pouring duration per bag, *t _pouring_* (min)	2 min	Measured as range for start of the pouring to changing the bag to new.
Emission rate, *G* (mg/min)	Triangular distribution with: Min 0.95 mg/min Mode 1.90 mg/min Max 3.80 mg/min	See description for Task 1 emission source.
Pouring time (emission time), *t _G_* (min)	2 min	The emission is assumed to continue through the whole repetition.
Task 3: Pouring 17×25 kg Micro Mica from small bags		
Parameter	Value	Comments
Repetitions per shift, *n* (-)	17	Seventeen small bags. Residual is not included.
Pouring duration per bag, *t _pouring_* (min)	Linear range: 1 to 2 min	Measured as range for start of the pouring to changing the bag to new.
Emission rate, *G* (mg/min)	Triangular distribution with: Min 1.85 mg/min Mode 3.7 mg/min Max 7.4 mg/min	See description for Task 1 emission source.
Pouring time (emission time), *t _G_* (min)	1 min	The emission is assumed to continue for 1 min.
Task 4: Simulated cleaning		
Parameter	Value	Comments
Repetitions per shift, *n* (-)	1	This is a simulated scenario to show the residual exposure from Task 3 powder pouring. This can occur when the worker prepares the pouring site for the next task. Emissions are not assumed to occur from cleaning.
Pouring duration per bag, *t _pouring_* (min)	10 min	
Emission rate, *G* (mg/min)	0 mg/min	
Pouring time (emission time), *t _G_* (min)	10 min	

The simulation results are reported as Geometric Mean (GM) concentration and Geometric Standard Deviation (GSD) and 5 ^th^, 95 ^th^ and 99 ^th^ percentiles. In a probabilistic exposure assessment, the predicted exposure risk is considered well-controlled when a 95 ^th^ percentile of the exposure concentration distribution is below 10% of the OEL when using a properly applied exposure model ( Hewett *et al*., 2006).

## Results

### Air mixing flow rate between NF and FF, *β*

The *β* calculated from concentration peaks 1 to 4 ( Figure 2) resulted to an average decay rate of 1405 min ^-1^ with standard deviation of 241 m ^3^/min. Assuming that NF volume is a cube with 1 m side length the free surface area for an open cube is 6 m ^2^ and for the pouring station (bottom, back, and both sides closed) it would be 2 m ^2^. The NF volume flow rate is 1405 m ^3^/min and the average random air velocity according to Eq. (S3) is ~470 m/min and ~1400 m/min for open and partially closed cubes, respectively. These are very high mean random air speed velocities for indoor environment as measured in following scenarios:

- In naturally ventilated industrial building with heat sources: 18 to 90 m/min ( Wang *et al*., 2016)
- 55 work areas within 27 different factories: geometric mean (GM) 3.6 m/min with geometric standard deviation (GSD) 1.96 ( Baldwin & Maynard, 1998).
- 16 workers in six indoor workplaces: average 12 m/min, range 6 to 94 m/min ( Berry & Froude, 1989).

Based on the observations where wind draft was not felt, the mean random air velocities estimated from the NF concentration decays are highly overestimated. This can be caused by incomplete mixing or overestimated NF volume. Because approximation method was not suitable for assessing the mean random air speed it was decided to use random air speed measurement data by Baldwin & Maynard (1998). For precautionary reasons, their office and school room measurement data were used; The lower air mixing increases the NF concentration where the worker is assumed to be during the work shift ( Table 1).

Eight ES with different CoU are modelled ( Table 2): the observed ES (no. 1), four precautionary scenarios (no. 2 to 5), one scenario with LEV (no. 6), one scenario with large room volume (no. 7) and a worst-case scenario including all precautionary actions (no. 8). These eight ESs covers all relevant combinations of CoU. The paint batch manufacturing time depends on the formulation recipe, which was here 150 minutes. Concentrations during the 150 min work shift varied from 0.08 to 1.57 mg/m ^3^ ( Table 2), corresponding to 8-h Time Weighted Average (TWA) concentration of 0.03 to 0.48 mg/m ^3^. Exposure distribution was narrow with GSD of 1.13 to 1.15 ( Table 2) and the 95 ^th^ and 99 ^th^ percentiles were on average 22.1% and 32.6% higher than the GM concentration. Simulation reports are given in Appendix S2 to S9 in *Underlying data* ( Koivisto *et al*., 2021), which show individual model parametrization, exposure distributions, exposure statistics, job sensitivity analyses and a random day concentrations in the NF. Task sensitivity analysis was performed only for the tasks in the observed scenario (no. 1).

**Table 2.** Near-Field (NF) concentration levels as geometric mean (GM) and geometric standard deviation (GSD), 95 ^th^ percentile and normalized with observed scenario GM concentration. AER is Air Exchange Rate. The exposure time is *ca*. 150 min. See Appendix S2 to S9 in *Underlying data* ( Koivisto *et al*., 2021) for the exposure distributions, a random day concentration, and job sensitivity analysis.

No. / scenario	GM, [mg/m ^3^]	GSD	95 ^th^ percentile, [mg/m ^3^]	GM normalized with scenario no. 1
**1.** Observed scenario	0.30	1.13	0.37	1.00
**2.** *G* range increased by ×2	0.51	1.14	0.64	1.71
**3.** *β* decreased by ×2	0.55	1.13	0.67	1.83
**4.** *AER* reduced by ×5 to 0.4 to 1.6 1/h	0.32	1.13	0.37	1.05
**5.** A small room ( *V _room_* = 100 m ^3^)	0.43	1.15	0.53	1.42
**6.** NF including LEV at 9.6 m ^3^/min	0.08	1.13	0.39	0.28
**7.** A large room ( *V _room_* = 10,000 m ^3^)	0.29	1.13	0.36	0.97
**8.** Worst-case: *G* increased, *β* decreased, *AER* decreased and small room ( *V _room_* = 100 m ^3^)	1.57	1.13	1.92	5.23

### The observed exposure scenario (no. 1)

**Figure 3.**
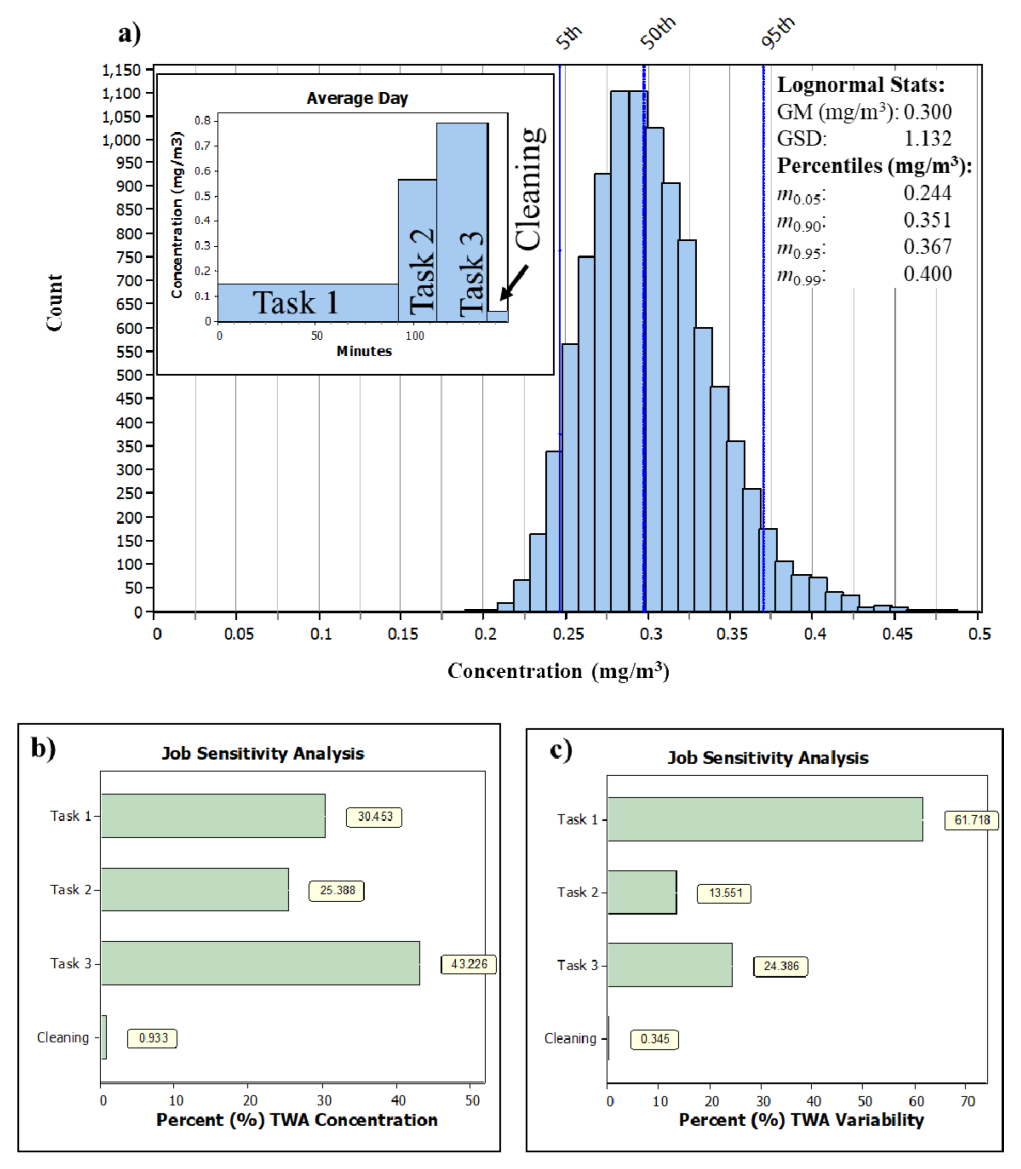
Simulation results showing **a**) exposure profile of the randomly generated concentration values and the task average concentrations for the N simulated work shifts (N = 10,000), where GM is geometric mean and GSD is geometric standard deviation, **b**) percent of average concentration and **c**) overall variability due to each task, respectively. TWA is Time Weighted Average. Sub-plot in **a**) shows the task-specific concentration levels. Tasks were 1. Pouring 4×500 kg TiO2 from big bags, 2. Pouring 10×25 kg TiO2 from small bags, 3. Pouring 17×25 kg Micro Mica from small bags, and 4. Simulated cleaning.

The GM NF concentration in the observed scenario was 0.30 mg/m ^3^ (0.24 and 0.37 mg/m ^3^ as 5 ^th^ and 95 ^th^ percentiles) with average task 1, 2, 3, and 4 GM concentrations of 0.15, 0.57, 0.79 and 0.04 mg/m ^3^, respectively ( Figure 3a). 8-h TWA GM concentration for TiO _2_, Micro Mica and inorganic dust were 0.05, 0.04 and 0.09 mg/m ^3^, respectively.

Job sensitivity analysis shows the contribution of different tasks to the work shift concentration ( Figure 3b). The contribution of Task 1, 2, 3 and 4 to the 8-h TWA as inorganic dust shift concentration was 30%, 25%, 43% and 1%, respectively ( Figure 3b). The greatest variability to work shift concentration level was attributed to Task 1 (62%) followed by Task 3 (24%) and Task 2 (14%) ( Figure 3c).

**Figure 4.**
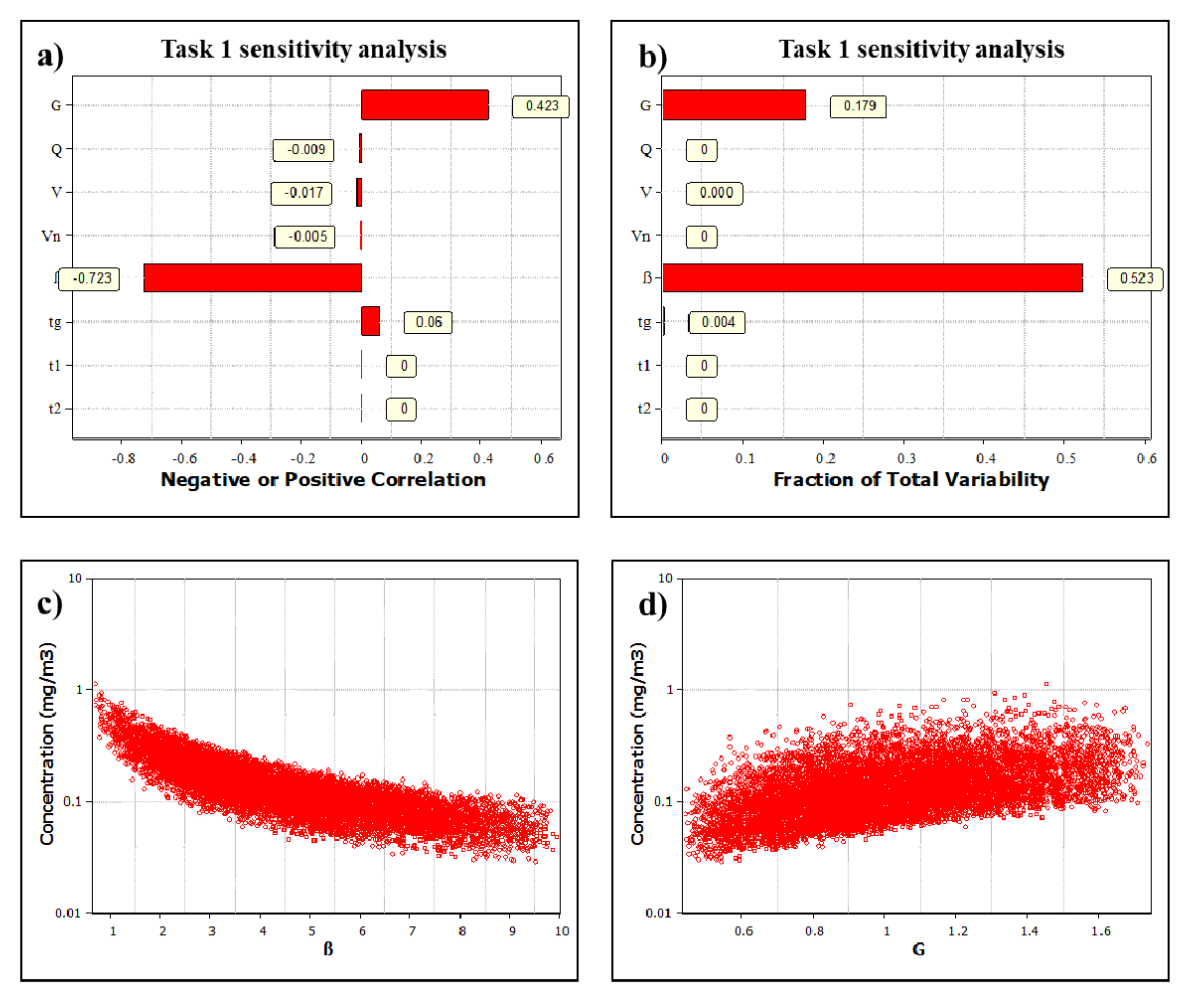
Main results from Task 1 sensitivity analysis: **a**) Spearman correlation coefficients showing if the exposure determinant effect on exposure is positive or negative and **b**) total variability showing how the exposure determinants cause the variation in the simulated exposure level. Figure **c**) and **d**) show the concentration relation with the air mixing flow rate between Near-Field (NF) and Far-Field (FF) (β) and generation rate (G), respectively.

Task 1 sensitivity analysis showed the relation of exposure determinants to the task concentration ( Figure 4). The emission rate ( *G*, mg/min) had a positive correlation of 0.42 and the air mixing flow rate between NF and FF ( *β*, m ^3^/min) had a negative correlation of -0.72. Similar results were found for Tasks 2 and 3 (data not shown). The variability in general ventilation does not have a significant influence on NF concentration level even when the variation is high ranging from 2 to 8 1/h. Room volume also has an insignificant influence on the NF concentration, but the variation is low, ranging from 1300 to 1500 m ^3^.

### Precautionary approaches (no. 2 to 5)

Parametrization in chemical safety assessment should follow precautionary principles favoring higher exposure estimates. Task sensitivity analysis showed that *G* has a considerable positive correlation and *β* has a strong negative correlation to the NF concentration level. It should be noted that the emission rate values were assigned using a conservative or precautionary approach that produces higher emission rate estimates than predicted by using more realistic NF parametrization (Text S3, Supporting Information). The impact of the following precautionary parametrization to NF concentration level were assessed:

- Increasing the range of minimum and maximum emission rate multipliers from 0.5 to 1 and 2.0 to 4.0, respectively, increases the GM concentration by 71% to 0.51 mg/m ^3^.
- Reducing *β* to half, *i.e.*, triangular distribution mode, minimum and maximum values are 1.6, 0.33 and 5 m ^3^/min, respectively, (mode was kept the same) increases the GM concentration by 83% to 0.55 mg/m ^3^.
- Reducing the general ventilation by a factor of 5 (0.4 ≤ *AER* ≤ 1.6 1/h) increases the GM concentration by 5% to 0.32 mg/m ^3^.
- Enclosing the pouring station into a 100 m ^3^ room (2 ≤ *AER* ≤ 8 1/h) increases the GM concentration by 43% to 0.43 mg/m ^3^.

### Local exhaust ventilation (no. 6)

The effect of the LEV rim along three sides of the pouring inlet was studied by applying a LEV to the NF. When pouring station LEV was operating, the flow rate was 9.6 m ^3^/min ( Fonseca *et al*., 2021). LEV emission efficiency was not reported but the particle number concentration measured from the LEV exhaust was at similar level as measured in the NF. Thus, it is assumed that the capturing efficiency is 0% and the LEV acts only as an additional ventilation exhaust in the NF. Model results showed that the LEV decreases the GM concentration by 72% to 0.08 mg/m ^3^.

### Increased room volume (no. 7)

Increasing the room volume to 10,000 m ^3^ (2 ≤ *AER* ≤ 8 1/h) decreases the GM concentration only by 3% to 0.29 mg/m ^3^.

### Worst case scenario (no. 8)

Worst-case parametrization by using the precautionary conditions no. 2 to 5 ( *i.e.*, 1× *G _mean_* ≤ *G* ≤ 4× *G _mean_*, *β* triangular distribution with mode, minimum and maximum of 1.6, 0.325 and 5 m ^3^/min, *V _room_* = 100 m ^3^, 0.4 ≤ *AER* ≤ 1.6 1/h) increases GM concentration by 423% to 1.57 mg/m ^3^ (1.92 mg/m ^3^ 95 ^th^ percentile), with average task 1, 2, 3, and 4 GM concentrations of 0.79, 2.46, 3.82 and 1.76 mg/m ^3^, respectively.

## Discussion

According to authors knowledge, this is the first study applying a probabilistic exposure model to specify safe CoU for an industrial process. The method is applicable when process emission rates are characterized at reasonable accuracy and the relevant exposure determinants are appropriately identified and quantified. Process emission rates are rarely reported, and this limits the use of probabilistic exposure assessment for estimating exposures. If process particle emission rates are not available, they can be estimated by using a precautionary approach such as a worst-case assumption - all used material becomes airborne (e.g., in sanding) or assuming that all process losses are emitted to air if process efficiency is known. In some cases, a concept of dustiness index for powders (mg/kg) can be useful to estimate emission rates ( Koivisto *et al*., 2015; Levin *et al*., 2014; Schneider & Jensen, 2009; Shandilya *et al*., 2019), but this should be used with precaution because it is a relative term describing the potential for dust emissions when the bulk material is handled or processed ( EN-15051; EN-17199). Also the powders are conditioned at 50% relative humidity before testing what can underestimate the powder dustiness in dry conditions ( Levin *et al*., 2015).

### NF concentration vs. personal exposure

The measured mean personal exposure level over the work shift was 0.52 mg/m ^3^ ( Koivisto *et al*., 2015), which is 3 times higher than the NF PM _4_ concentration of 0.17 mg/m ^3^ ( Figure 2). The following causes were identified to explain the difference between the NF PM _4_ and the PM _4_ personal exposure measurements:

- Koivisto *et al*. (2015) and Fonseca *et al*. (2021) identified the folding of empty bags as a source for increasing worker personal exposure level concentrations.
- NF sampling performed from the side of the worker and not between the source and the worker may not represent the breathing zone concentration level.
- The worker may have been exposed to other sources such as forklift resuspension dusts or emissions from scaling the residual powder.

The differences may also be based on the fact that ( Parlar & Greim, 2005):

- employees do not remain in the same place for the entire duration of sampling,
- the stationary sampling device can be obstructed by the employee,
- the concentration depends on the distance from the emission source, and
- the local concentration is influenced by air currents.

It has been shown that stationary and personal sample populations exhibit a GSD ranging from 1.4 to 2.6 ( Harrison, 2002), but the relation should be evaluated carefully depending on the pollutant type, other emission sources, and work tasks ( Cherrie, 2004; Lange, 2003; Westerlund *et al*., 2019). Stationary measurements alone are not sufficient for personal exposure assessment. The correlation between personal and stationary samples should be evaluated. This also helps to recognize if the underlying exposure determinants are properly understood, such as background sources of the pollutants.

### Predictive exposure assessment

In the observed scenario (no. 1), the predicted concentration level was 0.30 mg/m ^3^, which is clearly higher than the measured NF concentration of 0.17 mg/m ^3^ ( Koivisto *et al*., 2015). The difference between measured and modelled concentration is explained by the emission rate, that was adopted from Koivisto *et al*. (2015), who used 8 m ^3^ NF volume and 10 m ^3^/min flow rate between the NF and FF (Text S3, Supporting Information), which produces higher estimates for the emission rate. This can be considered a precautionary emission rate assessment that is recommended if dispersion and dilution are not well known.

The personal exposure level over the work shift was 0.52 mg/m ^3^, which is clearly higher than the predicted GM concentration of 0.30 mg/m ^3^ and the 95 ^th^ percentile of 0.40 mg/m ^3^. This is mainly caused by the difference between NF and personal exposure PM _4_ measurements.

In the worst-case scenario, Tasks 1, 2, 3 and 4 average concentrations are 0.79, 2.46, 3.82 and 1.76 mg/m ^3^ with task durations of 92, 20, 26 and 10 min, respectively. This would correspond to 8-h TWA GM concentration for TiO _2_, Micro Mica and inorganic dust (both TiO _2_ and Micro mica) of 0.26, 0.21 and 0.48 mg/m ^3^, respectively. If the 95 ^th^ percentile is used, the respective concentrations are ca. 22% higher. The occupational exposure limit values OELVs as 8-h TWA concentrations are 10 mg/m ^3^ for TiO _2_ and 5 mg/m ^3 ^for Micro Mica (inorganic dust), which are 39 and 24 times higher than the respective modelled 8-h TWA GM concentrations. The 95 ^th^ percentile 8-h TWA work shift concentration would be 0.59 mg/m ^3^ that is 8 times lower than the OELV for inorganic dust. Here the sampling cassette wall losses were not taken into account, and this reduces the OLEV exposure-ratio. It is good to note that in a 100 m ^3^ room the work is challenged by the limiting space and this value should be considered as an over precautionary estimate for a reasonable worst-case room volume.

It can be concluded that paint batch manufacturing is safe under RWC conditions. If production is scaled up to two paint batches per day, it would double the 95 ^th^ percentile of 8-h TWA exposure and reduce exposure to OELV ratio to 4. In this case, it would be recommended to add a LEV in the pouring station. The personal exposure level in the worst-case scenario should be verified with personal sampling.

### Job sensitivity analysis

Job sensitivity analysis showed that the best approach to reduce exposure is to reduce Task 3 concentrations followed by Task 1, 2 and 4 ( Figure 3b). The best approach to reduce the model variability ( *i.e.*, GSD) is by reducing Task 1 variability ( Figure 3c).

Here, Task 2, 3, and 4 concentrations do not separate residual concentration tails, meaning that a small fraction of TiO _2_ is mixed with Micro Mica. Residual concentrations without other emissions were evaluated in the simulated cleaning part with the job sensitivity analysis (Appendix S2 to S9, *Underlying data* ( Koivisto *et al*., 2021)). The contribution of Task 4 to 8-h TWA concentration varied from 0.2% (no. 6) to 11% (no. 8) where the Micro Mica and inorganic dust 8-h TWA concentrations would be underestimated by 18% and 8%, respectively, if the residual concentration in the cleaning is ignored. The uncertainty can be reduced by adding time between the pouring processes between Task 1 and 2, as shown in the random day concentrations (Appendix S2 to S9, *Underlying data* ( Koivisto *et al*., 2021)).

### Exposure determinant analysis

For NF concentration, the most relevant exposure determinants were the emission rate and the air mixing between the NF and FF ( Table 2; Figure 4). Room volume, NF volume and general ventilation AER were factors that were significantly less relevant for NF exposure level. With the same AER (2 to 8 1/h), a 15 times smaller room ( *V _room_* = 100 m ^3^) increased the NF concentration only by 43%, and a 6.7 times larger room ( *V _room_* = 10,000 m ^3^) decreased the NF concentration by only 3%. Reducing the pouring hall air exchange rate by a factor of 5 increased NF concentration by only 5%. According to the modeling, a LEV as an additional exhaust in the NF is an efficient method to reduce the NF concentration levels. Unfortunately, Fonseca *et al*. (2021) do not report NF respirable mass concentration for comparison.

This demonstrates that the *G* and *β* are the most influential exposure determinants for the NF concentration. The model accuracy can be significantly improved with better quantification of these determinants. Here, the NF volume was small as it considered the emission source distance from the worker to be 50 to 65 cm and the partial closure was included in the model. Thus, modeling results can be considered sufficiently accurate for exposure and risk assessment regardless if NF volume, room volume or general ventilation are precisely known. It can be stated that the above factors are not relevant for risk communication reducing the number of OCs needed to evaluate CoU. It would also be beneficial to identify the background sources with a real time personal monitor and add this contribution to the measured personal exposure concentration. 

It is helpful to note that in task sensitivity analysis ( Figure 4) a variable with a small range or constant value does not contribute to the variability, but will contribute to the magnitude of the estimates, as demonstrated with different CoU modeling results ( Table 1). However, some exposure determinants are well specified, such as pouring times and powder amounts by the paint formulation and batch volume, and do not affect model prediction range. The task sensitivity assessment does not include systematic errors.

### Compliance with REACH regulation

REACH legislation requires deriving quantitative exposure estimates for all contributing scenarios to support the risk characterisation for materials for which Derived No Effect Limits or Derived Minimum Effect Limits have been determined. Exposure estimates can be based on modelling tools and/or on measured data sets. One of the prerequisites to be ensured using models is that the conditions described in the exposure scenario are consistent with the applicability domain of the modelling tool. The applicability domain is quite limited in the case of empirical models, as it reflects the observed data ranges and scenario concepts used to parameterize the empirical model parameters. On the contrary, the applicability of a mass balance model spans to any magnitude of the determinants and it is only limited to the modelling concept. For instance, any emission rate, room volume, LEV, *etc.* can be modelled with the NF/FF model, with the accuracy ensured by applying physics laws in a mechanistic setting. On the other hand, a multi-source industrial setting usually requires a multi-compartment model.

REACH legislation implemented by ECHA requires no risk assessment in cases of absence of exposure or hazard, but it explicitly states that it is unlikely that exposure modelling alone will provide the level of proof to demonstrate highly controlled conditions ( ECHA R.14). Nonetheless, ECHA R.14 recognises that when managed effectively, enclosure, containment and process ventilation have the potential to prevent releases and only higher tier models allow assessment of these types of circumstances. Using the tier 2 mass balance modelling methodology, proposed here, captures in detail the sensitivity of the concentration to the exposure determinants and allows the ability to define which scenario ( *i.e.*, interventions in which determinant) is closer to negligible exposure conditions.

Another issue tackled by the NF/FF model that legislation underlines is how activity-specific exposure must be estimated. The workers must not be exposed to activity specific levels for the times not performing the task and the two-zones model provides both this, even crude, spatial distinction together with a reasonable ( *e.g.* 1 min) temporal resolution. The latter provides a refinement to the assessment that allows for determining acute exposure as well. Legislation guidance stresses the need for accurate modelling of the temporal dynamic of peak concentrations of exposure expected due to the nature of the activity, in contrast with stable exposure levels, that can be extrapolated from long-term estimations.

Legislation requires that exposure scenarios assessed must cover all the described uses and take into account the variability within and between tasks, and for users and sites. The modelling shown here can incorporate several tasks in one job simulation and quantify the impact of each task on the overall job exposure. Regarding variability, it can be attributed to a number of factors, including differences in the application of OCs, level of (substance) throughput, other local conditions, variability in performance of RMMs, (lack of) maintenance of plant over time, behavioural differences between workers, *etc.* Variability is accounted in by using the probabilistic features of the model and assigning realistically wide distributions for exposure determinants. The estimated concentrations are given in percentiles, as suggested by the legislator, to allow for case scenario determination *(e.g.* the 90 ^th^ percentile used for the worst-case scenario typically in REACH).

### Compliance with EN 689 strategy for testing compliance with OEL values

According to EN 689: 2018+AC:2019 a basic exposure characterization is needed to decide if personal exposure measurements are needed. This can be obtained by using adequate exposure models. The exposure assessment methodology applied here for the powder pouring fulfils the transparency requirements. The different exposure modelling results are based on quantitative exposure determinants and the link with personal exposure was established. Based on the assessment, there is no need for further personal exposure measurements.

Personal exposure measurements are needed if the basic exposure characterization shows non-compliance. The compliance can be statistically evaluated by comparing the OELV with the upper confidence limit (UCL) of 70% with the 95 ^th^ percentile of the distribution of at least six measurements in Similar Exposure Group (SEG). If the UCL is lower than the OELV, it is concluded that the probability of exceeding the OELV is acceptably low: the decision is compliance. After adequate CoU have been achieved, reassessment of the situations should be conducted on a regular basis in order to assess if the exposure remains stable over time ( EN 689, Appendix I). The test requires three to five exposure measurements on workers belonging to a SEG, depending if the results are below:

1) 0.1 OELV for a set of three exposure measurements or,
2) 0.15 OELV for a set of four exposure measurements or,
3) 0.2 OELV for a set of five exposure measurements

then it is considered that the OELV is respected and complies with EN 689. If compliance is inconclusive, the situation requires additional measurements. If compliance is concluded, the measurements should be repeated as:

- GM < 0.1 OELV 36 months,
- 0.1 OELV < GM < 0.25 OELV 24 months,
- 0.25 OELV < GM < 0.5 OELV 18 months,
- 0.5 OELV < GM 12 months.

Thus, it is recommended setting the CoU at the level where GM exposure level is below 0.1 OEL to minimize the need for exposure measurements.

In this case, the observed scenario (no. 1) is expected to meet the requirements for 36 months measurement period. According to our knowledge, the compliance should be evaluated by revising that the OCs meets the CoU criteria established in the basic exposure characterization. Here, the worst-case exposure scenario (no. 8) compliance should be verified with personal measurements because the 95 ^th^ percentile was 0.125 of the OELV. In this case, the basic assessment precision can be significantly improved if the air mixing between the NF and FF is better characterized *e.g.*, by measuring the random air flow velocity under different CoU. In general, it is recommended to perform random air flow velocity and turbulence rate measurements *e.g.* by using a multidirectional hot wire anemometer when characterizing the process emissions.

### Applications beyond REACH exposure assessment

Finally, it is important to point out the usefulness and applicability of exposure models in other fields of application, in addition to the one described in this paper for REACH ( EC, 2014). In the area of industrial process, where other existing product and use regulations also apply, such as for example the Machinery Directive 2006/42/EC and the EHS Directives ( EC, 2016; EC, 2019), this modelling approach can replace/complement traditional emission/exposure measurement approaches, in various stages of the life cycle of processes.

The first application relates to the design of new (non-existent) industrial manufacturing processes and equipment (e.g. machinery and assemblies of machinery). In this case, it is necessary to assess the risks derived from potential emissions of hazardous materials and substances originating from the process and mitigate them by design ( EN 1093-1; EN ISO 12100; EN ISO 14123-1), in order to demonstrate the conformity with the mandatory essential health and safety requirements (EHSR). Thus, such models can be useful tools to deploy a safe and sustainable-by-design approach in the design of new industrial production processes ( EC, 2020). 

A second application connected with the previous one, refers to the verification of the effectiveness of the protective measures for risk prevention and/or reduction from airborne emissions/exposures, implemented or to be implemented in industrial processes and workplaces (e.g. LEVs, enclosures, etc) ( Mulhausen, 2009).

A third area of applicability lies in the assessment and periodic monitoring of the occupational exposure of workers to chemical agents at work, in order to demonstrate compliance with mandatory/voluntary OELs ( EN 689).

Finally, within the framework of the digital transition currently facing European industry ( EC, 2021), digital technologies such as smart sensors, artificial intelligence, internet of things or big data, can also play an important role in reducing the overall environmental footprint of manufacturing processes ( EC, 2020). In this context, exposure models can be part of the architecture of more complex models, integrated into advanced digital tools such as Digital Twins, aimed at optimizing manufacturing processes in real time with the purpose, among other functionalities, of preventing, predicting and reducing impacts on human health and the environment, and in our particular case, emissions and occupational exposures to chemical agents.

## Conclusions

Here we have explained the benefits of exposure assessment based on regional concentration levels and the data requirements for predicting personal exposures in different conditions of use. We identified relevant exposure determinants for predictive exposure assessment. The methods were demonstrated by applying a probabilistic NF/FF exposure model to estimate worker exposure to respirable particles during pouring of pigment and filler powders. The probabilistic NF/FF model was used to identify sensitive pouring tasks and the importance of exposure determinants having an impact on the exposure level. The sensitivity analysis consisted of two parts:

- Job sensitivity analysis, which was used to calculate the contribution of different tasks to the work shift personal exposure level.
- Task sensitivity analysis, which was used to identify relevant exposure determinants and their impact on concentration level in each task.

Predictive exposure assessment was performed for different powder pouring scenarios. Precautionary and worst-case scenario parametrization revealed that the exposure levels during paint batch manufacturing is safe, but the conclusion needs to be revised if production is scaled up to two paint batches per day. It was demonstrated that if sensitive exposure determinants are sufficiently well known it is possible to predict the work shift exposure with acceptable precision regardless of precautionary assignment of non-sensitive parameters.

Probabilistic exposure assessment is an efficient method to identify relevant tasks considering worker exposure. Identification of sensitive exposure determinants helps to find efficient emission and exposure control techniques and their impact on exposure level can be simulated before implementing the changes. Personal exposure measurements are needed to validate the model and to monitor the borderline scenarios where the predicted exposure 95 ^th^ percentile is >0.1 × limit value. Improvement of the modeling parameters such as the level NF ventilation will increase the value of the model and potentially reduce the need for monitoring.

## Data availability

### Underlying data

Zenodo: SUPPLEMENTARY MATERIAL for Assessment of exposure determinants and exposure levels by using stationary concentration measurements and a probabilistic Near-Field/Far-Field exposure model. http://doi.org/10.5281/zenodo.4905515 ( Koivisto *et al*., 2021).

This project contains the following underlying data:

- Supplementary material 7 June.docx (Text S4. Air mixing flow rate between NF and FF, β; Appendix S1 to S9 simulation reports).

### Extended data

Zenodo: SUPPLEMENTARY MATERIAL for Assessment of exposure determinants and exposure levels by using stationary concentration measurements and a probabilistic Near-Field/Far-Field exposure model. http://doi.org/10.5281/zenodo.4905515 ( Koivisto *et al*., 2021).

This project contains the following extended data:

Supplementary material 7 June.docx (Text S1. Terminology according to ECHA R.14; Text S2. Data requirements for exposure modeling and emission source characterization; Text S3. Generation rate)

Data are available under the terms of the Creative Commons Attribution 4.0 International license (CC-BY 4.0).

## List of abbreviations

**Table 3.** 

CoU	Conditions of Use	OC	Operational Condition
CSs	Contributing Scenarios	OELV	Occupational Exposure Limit Value
ECHA	European Chemicals Agency	REACH	Registration, Evaluation, Authorisation and Restriction of Chemicals
ES	Exposure Scenario	RMMs	Risk Management Measures
FF	Far-Field	RWC	Reasonable Worst Case
GM	Geometric Mean	SEG	Similar Exposure Group
GSD	Geometric Standard Deviation	TWA	Time Weighted Average
NF	Near-Field	EHSR	Environmental Health and Safety Requirement
