# Supplementary material for: Assessment of exposure determinants and exposure levels by using stationary concentration measurements and a probabilistic near-field/far-field exposure model
Source: Open Res Eur. 2021 Jun 21;1:72. [Version 1] doi: 10.12688/openreseurope.13752.1 (PMC10446057; doi:10.12688/openreseurope.13752.1)
Supplement: Peer review history

# Peer review: Reviewer response for version 1

**DOI:** 10.21956/openreseurope.14830.r28272

**Published:** 2022-01-26

**Authors:** Thomas W. Armstrong

**Affiliations:** 1 TWA8HR Occupational Hygiene Consulting LLC, Branchburg, NJ, USA

The project's stated goals of using appropriate modeling to evaluate conditions of use, the effects of determinants of exposure, and compare those results to available personal and area concentration data are well met and clearly described in the manuscript. The use of Monte Carlo simulations to explore the sensitivity of the model results to changes in the determinants of exposure is both appropriate and clearly described. This notable contribution demonstrates the utility of the near-field, far-field mass balance model in evaluating the impact of changes in determinants of exposure including mass emission rate, general ventilation, and near-field, far-field air exchange on the magnitude of potential exposure due to changing conditions.  

 A few minor concerns identified are:

1. Page 6, first paragraph, last sentence. The meaning of this sentence is not clear. Please explain what you are stating in a bit more detail.
2. Page 12, column two, top paragraph, second line. Suggest "testing, and that may" rather than "testing what can..."
3. Page 12, column 2, last paragraph "Predictive exposure assessment".  Yes, the predicted 0.3 mg/m3 is higher than the measured NF concentration of 0.17 mg/m3. However, in the world of realistic worker and environmental variability, these are very similar and could be from the same exposure distribution.  Somehow, the variability reported could be lower than expected for a task that has several components and is conducted at a workstation that does not have rigorous control over local air exchange, air current directions, general ventilation, and contaminant generation. Perhaps this aspect (limited variability in the studied scenario) could deserve mention in the Discussion section. 

Is the study design appropriate and does the work have academic merit?

Yes

Is the work clearly and accurately presented and does it cite the current literature?

Yes

If applicable, is the statistical analysis and its interpretation appropriate?

Yes

Are all the source data underlying the results available to ensure full reproducibility?

Yes

Are the conclusions drawn adequately supported by the results?

Yes

Are sufficient details of methods and analysis provided to allow replication by others?

Yes

Reviewer Expertise:

Exposure assessment using mathematical models, determinants of exposure, Monte Carlo simulation, statistical analysis of workplace expsoure data,

I confirm that I have read this submission and believe that I have an appropriate level of expertise to confirm that it is of an acceptable scientific standard.

# Peer review: Reviewer response for version 1

**DOI:** 10.21956/openreseurope.14830.r28021

**Published:** 2021-12-07

**Authors:** Paul Price

**Affiliations:** 1 National Exposure Research Laboratory, Office of Research and Development, United States Environmental Protection Agency, Research Triangle Park, NC, USA

Under REACH, the need for accountability in assuring the safety of workers and the concurrent need for assurance that a workplace is in compliance has led to a system of exposure scenarios, contributing scenarios, and operating conditions. In order use this method, a new exposure assessment may be required whenever there is a change in any one of the relevant operating conditions. The paper proposes an approach that seeks to avoid this problem by identifying the subset of operating conditions that drive variation in dose and to identify the range of values those parameters could plausibly take. Such an analysis allows the analyst to demonstrate that the task would be safe for all reasonable expected exposures even if the exact values for the inputs are not known. This approach is demonstrated using two example chemicals and a specific exposure scenario that have existing monitoring and modeling data available.

 The purpose of the paper, the materials used in the paper, and the approach used in the paper appear reasonable and appropriate. The use of an existing data set allowed the paper to focus on the methodology for determining safety and not become bogged down in describing the generation of the monitoring data and modeling inputs.

 Specific comments

1. Page 6. First paragraph. Last sentence. The current text reads “In this study, the losses were not taken into account because it should be evaluated by using the occupational aerosol.” It is not clear what is meant by this text. Not accounting for these losses would result in underestimates of dust concentrations in the measurements.
2. Page 6. Move the CAS number for the two chemicals to the first time they are cited in the paper on page 5.
3. In the supplemental data, the plots of the random day concentrations have four distinct peaks followed by a forest of large but short-term peak exposures. Could you explain the plots in terms of the four tasks and the exposure models? What behaviors do the four peaks correspond to and why is there a forest of large but short-term peak exposures after 90 minutes?

Is the study design appropriate and does the work have academic merit?

Yes

Is the work clearly and accurately presented and does it cite the current literature?

Yes

If applicable, is the statistical analysis and its interpretation appropriate?

Yes

Are all the source data underlying the results available to ensure full reproducibility?

Yes

Are the conclusions drawn adequately supported by the results?

Yes

Are sufficient details of methods and analysis provided to allow replication by others?

Yes

Reviewer Expertise:

Exposure assessment, modeling interindividual variation in exposure, use of probabilistic models, integrating science and policy in regulatory affairs.

I confirm that I have read this submission and believe that I have an appropriate level of expertise to confirm that it is of an acceptable scientific standard.
